# Learned interval time facilitates associate memory retrieval

**DOI:** 10.1101/lm.044404.116

**Published:** 2017-04

**Authors:** Vincent van de Ven, Sarah Kochs, Fren Smulders, Peter De Weerd

**Affiliations:** Department of Cognitive Neuroscience, Faculty of Psychology and Neuroscience, Maastricht University, Maastricht 6200 MD, The Netherlands

## Abstract

The extent to which time is represented in memory remains underinvestigated. We designed a time paired associate task (TPAT) in which participants implicitly learned cue–time–target associations between cue–target pairs and specific cue–target intervals. During subsequent memory testing, participants showed increased accuracy of identifying matching cue–target pairs if the time interval during testing matched the implicitly learned interval. A control experiment showed that participants had no explicit knowledge about the cue–time associations. We suggest that “elapsed time” can act as a temporal mnemonic associate that can facilitate retrieval of events associated in memory.

An important contextual feature in memory formation and retrieval is time (Tulving 1985; Polyn and Kahana 2008; Eichenbaum 2014). For example, temporal order binds events in memory, such that participants recognize more items if they are presented in the same order in which they were learned (Schwartz et al. 2005; Tubridy and Davachi 2011; Hsieh and Ranganath 2015). The concept of time can also be studied as “elapsed duration,” which may also be stored as part of an event in episodic memory (Eichenbaum 2014). It is unclear how elapsed time is stored in memory, although some recent studies started to tackle this issue. Recent neurophysiological recordings in rats showed data consistent with the idea that medial temporal lobe (MTL) structures, including the hippocampus, code elapsed time (MacDonald et al. 2011). This finding is perhaps related to previous functional magnetic resonance imaging (FMRI) studies showing that the hippocampus uses temporal proximity to bind discontinuous visual events in memory (Schapiro et al. 2012; Hsieh et al. 2014). Yet, human behavioral studies about how elapsed time facilitates memory processes are largely lacking. Therefore, we designed a time paired association task (TPAT) in which participants implicitly learned associations between visual events and particular time intervals. Importantly, participants were not informed about the time intervals. We hypothesized higher accuracy for identifying matching visual events for those trials in which the time interval during testing matched the cue-dependent interval that was previously learned.

Participants (*N* = 41) completed the TPAT in Experiment 1 (see Supplemental Methods). We used colored abstract shapes (size = 5° × 5° visual angle; see Supplemental Methods and Supplemental Fig. S1) to minimize conceptual or semantic processing of participants. The shapes were presented as eight different stimulus pairs in which half of the shapes served as memory cues and the other half as memory targets in the TPAT. The task comprised three phases: a passive exposure phase, an active learning phase, and a test phase.

In the passive exposure phase, participants were shown the eight cue–target pairs once, with the target always shown after presentation of the cue. All stimuli were shown for 2000 msec. In half of the pairs, the target was shown 500 msec after cue offset (i.e., cue–target interval [CTI] of 500 msec). In the other half of the pairs, the CTI was 2000 msec. The order of the eight pairs was randomized for each participant. Only the to-be trained pairs were shown, and participants were encouraged to already start learning the associations between the stimuli, but did not have to make a response.

In the learning phase, participants trained their memory of the eight cue–target pairings. Each learning trial began with the presentation of a cue, which was followed by a CTI of 500 or 2000 msec (50% probability for either CTI), after which a “probe item” was shown (Fig. 1A,B). Probe items could represent either the target that was associated with the cue, or a nontarget. In the learning phase, the probe item was the target on 50% of the trials. Nontarget probes were randomly drawn from one of seven nontarget alternatives. Participants were required to judge whether the probe item was identical to the cue-associated target (two-alternative forced-choice [2AFC] judgment). Importantly, when the probe was a target, it was always shown at the CTI with which it was shown in the passive exposure phase. Nontarget probes were shown at the complementary time interval. Participants received trial-by-trial feedback about response accuracy, and feedback about their performance at the end of each block. The associations were practiced in blocks of 32 trials until reaching performance criterion of 84% correct, or up to a maximum of six learning blocks (participants completed on average 4.1 [median = 4, SD = 1.4] learning blocks). Each block of 32 trials presented each cue stimulus four times in random order.

**Figure 1.**
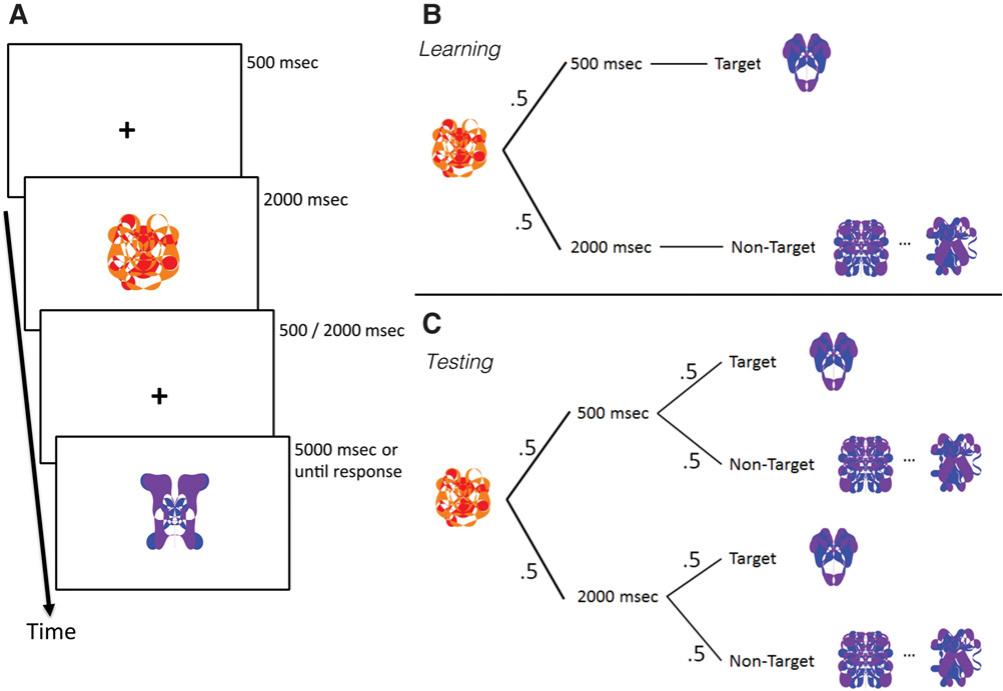
Time paired association task (TPAT) design. (*A*) Experimental design of a typical trial used in the learning and testing phases. (*B*,*C*) Schematic display of the probabilities of cue, time, and probe (target or nontarget) presentations in trials during the learning (*B*) and testing phases (*C*).

In the test phase (Fig. 1C), participants completed a number of test trials in a similar fashion as in the learning phase, but with two crucial differences. First, the cue–time–target contingency established during learning was broken, such that a target could be presented either at the learned CTI or at the other time interval at which the target never had been shown (see Fig. 1C). In test trials, the probe could be a nontarget (for which no timing information is learned) or a target. When the probe was a target (50% of all trials), the time interval used at test could match the learned CTI or not at equal probability. Hence, the most relevant aspect of our experimental design can be described as a 2 × 2 factorial design with as factors CTI used during prior learning (Learned CTI) and CTI used during testing (Tested CTI). A second difference with the training phase was that participants did not receive any feedback. Importantly, the 2AFC judgment was the same as in the learning phase. Trial order was randomized for each block (each block containing 64 trials) and participant.

Experiment 1 was run in two versions: Participants in Experiment 1a completed 64 testing trials (*N* = 17), whereas participants in Experiment 1b completed 256 testing trials (*N* = 24). Otherwise, Experiments 1a and 1b were identical in all respects. Analyses showed no differences between Experiments 1a and 1b (see Supplemental Results and Supplemental Table S1) and we therefore pooled the data between them.

The data were trimmed by removing trials with extremely fast and slow response times (cutoff at ±2 SD) and trials without a response in each participant, discarding ∼5% of trials on average across participants. The dependent variables sensitivity (*d*′, see Stanislaw and Todorov 1999; Macmillan and Creelman 2005) and response time (of correctly judged test trials only) were analyzed separately using a repeated-measures analysis of variance (RMANOVA) with factors Learned CTI (500, 2000) and Tested CTI (500, 2000) and the first-order Learned CTI × Tested CTI interaction term. We were mostly interested in the interaction term, as this effect best represents a memory bias based on learned cue-dependent time delays. We report effect sizes and post hoc pairwise comparisons where deemed appropriate.

Results showed that cue–target pairs were judged more accurately when the CTI during testing was the same as the CTI during learning (see Fig. 2A). RMANOVA of *d*′ as dependent variable revealed no significant main effects of Learned CTI or Tested CTI (see Supplemental Table S2), but a significant Learned CTI × Tested CTI interaction effect (*F*_(1,40)_ = 12.2, *P* = 0.001, ep^2 = 0.23). Post hoc comparisons showed higher *d*′ for cue–target pairs that were learned with a short CTI when these pairs were also tested with the short CTI (mean [SE] *d*′ = 2.4 [0.2]), compared with when tested with the long CTI (2.1 [0.2]; *t*_(40)_ = 2.5, *P* = 0.012, Cohen's *d* = 0.38). A similar effect was found when pairs learned with the long CTI were tested with the long (2.5 [0.2]) versus short interval (2.2 [0.2]; *t*_(40)_ = −2.9, *P* = 0.007, Cohen's *d* = 0.45). Thus, these results suggested that participants used knowledge of time to their benefit in recognizing cue–target pairs, such that test trials with congruent cue–target intervals were recognized with higher accuracy than trials with incongruent intervals.

**Figure 2.**
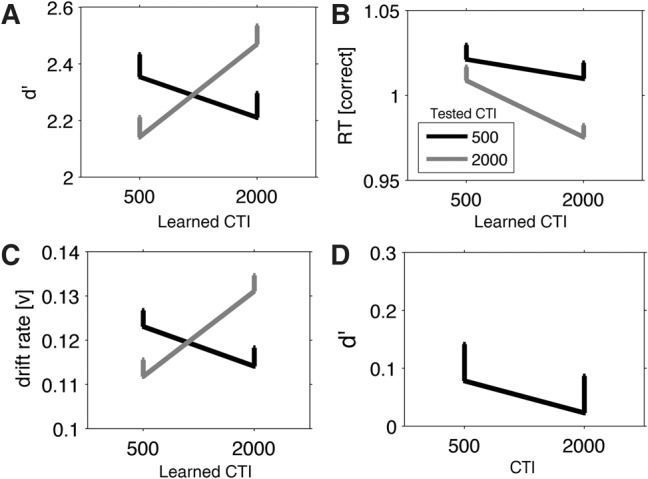
Results. Shown are the mean *d*′ (*A*), reaction times (*B*), and drift rates (*C*) for the four conditions of the Learned CTI and Tested CTI factors in Experiment 1. Black (gray) line represents accuracy for the tested cue–target interval (CTI) of 500 (2000) msec. Interaction effect was significant for accuracy and drift rate, but not for reaction times. (*D*) Mean recognition *d*′ of the short and long CTI in Experiment 2, which did not significantly differ from chance-level performance. RT[correct] = reaction times of hit trials; *v* = EZ-diffusion model drift rate parameter (see Supplemental Materials). Error bars represent 1 SEM.

Analysis of reaction times (see Fig. 2B) showed shorter response latencies for long test CTIs (mean [SE] RT = 1010.6 [71.8] msec), compared with short test CTIs (1029.8 [71.5] msec; main effect of Tested CTI, *F*_(1,40)_ = 6.4, *P* = 0.016, ep^2 = 0.14), which fits to the well-known response facilitation with longer foreperiods (Näätänen 1971; Niemi and Näätänen 1981; Los et al. 2014). The main effect of Learned CTI and the interaction effect were not significant (see Supplemental Table S2).

A potential complication in the TPAT is that the systematic variations in time interval could alter responses independently of memory-related effects, such that longer-lasting TPAT trials could lead to participants endorsing different response strategies for different temporal conditions. Separate analysis of response latencies and accuracy can provide an incomplete description and could thus obscure such confounding effects. To provide a more comprehensive description of TPAT performance, we used a diffusion model of decision-making, which combines distributions of response speed and accuracy to provide a latent variable description of decision-making parameters (Ratcliff 1978; Wagenmakers et al. 2007; Ratcliff and McKoon 2008; Voss et al. 2013). Analysis of the parameters of the EZ-diffusion model (Wagenmakers et al. 2007) showed that recognition decisions were made more easily for those trials in which the presented CTI matched the learned CTI (see Fig. 2C) (*F*_(1,40)_ = 15.1, *P* < 0.001, ep^2 = 0.27), without concomitant changes in “response bias” or “response delays” (see Supplemental Results), thereby corroborating the previous results.

Did participants have explicit knowledge about the cue-related time intervals during testing? To answer this question, we qualitatively assessed during debriefing whether participants were aware of the variations in time intervals across the cue–target pairs. Four participants reported that they had become aware of the variation in time delays and that they had used this knowledge during the test phase. Removing them from the analyses did not change the pattern or significance of the results, indicating that knowledge of time was generally implicit. Nevertheless, participants could have knowledge of the associated time intervals that might not be accessible during post hoc debriefing. To verify more objectively whether participants had knowledge of the time intervals, we designed a second experiment (*N* = 16) in which we tested whether the cue elicited a temporal expectation for the onset of a probe item. Again, participants were not informed about CTI associations, and completed a passive and learning phase that was identical to that of Experiment 1. Afterward, participants completed a time recognition task (TRT), in which a cue stimulus was followed by one of two time intervals after which the same cue stimulus was shown again. In contrast to the TPAT, in which participants had to respond to the second (probe) stimulus, the second stimulus in the TRT served to indicate the end of the time interval within the trial. Participants had to judge if the CTI was associated to the cue or not. The TRT contained two blocks of 64 trials each. For the analysis, we compared response times and accuracy of the recognition judgments between the two tested CTIs. A CTI for a given cue during the test phase was a Match trial if the CTI was the same as was learned for that cue, and a NonMatch trial otherwise.

Analysis (trimming at ±2 SD, discarding <5% of trials) showed no significant difference in response times between the short (1279.6 [190.6] msec) and long CTI (1218.5 [211.4] msec) during testing (*F*_(1,15)_ = 2.13, *P* = 0.17). Further, participants showed recognition *d*′ at chance level (one-sample *t*-test against chance level of *d*′ = 0) for the short (0.08 [0.1]; T[15] = 0.7, *P* = 0.51) and long CTI (0.02 [0.1] %; T[15] = 0.2, *P* = 0.87) during testing (see Fig. 2D), which did not differ between either CTI (*F*_(1,15)_ = 0.2, *P* = 0.67). A similar RMANOVA as for the TPAT experiment showed no significant main or interaction effects for response time or accuracy (see Supplemental Tables S3, S4). Thus, participants were largely unaware, or did not have explicit knowledge about the cue–time associations.

Our findings fit with the suggestion that temporal context can implicitly facilitate memory processes (Polyn and Kahana 2008; Eichenbaum 2014; Ranganath and Hsieh 2016). Previous studies showed that temporal order of discontinuous events (Tubridy and Davachi 2011; Hsieh et al. 2014) and temporal proximity between events (Schwartz et al. 2005; Schapiro et al. 2012) facilitate memory formation and retrieval. We extend these findings by showing that cue-dependent memory of elapsed time also facilitates retrieval of associated visual objects.

Further, our findings suggest some level of accuracy in time interval perception, which may in turn be related to memory (Los et al. 2014; Gu et al. 2015). For example, higher working memory capacity or efficiency could be associated to better duration judgments (for review, see Gu et al. 2015). Also, participants may form a short-lasting, implicit representation of time duration as a function of a distribution of trial durations in an experimental context (Dyjas et al. 2012; Los et al. 2014). Combining our results with these findings, it is conceivable that different time memories share common resources or representations, an intriguing suggestion that warrants further investigation.

Our results may be further related to studies showing that attentional resources can be temporally allocated (Coull and Nobre 2008). In these studies, a cue stimulus predicts the presentation of the target stimulus at a particular moment in the near future, thereby creating a temporal expectancy about when to allocate attention in order to optimize sensory processing (Correa et al. 2005; Martens and Johnson 2005; Rohenkohl et al. 2011, 2012; Vangkilde et al. 2012). Our study extends the notion of temporal cueing in several ways. While in previous studies the temporal cues were explicitly associated to time delays, cue–time associations were implicit in our study. Even more, time was irrelevant to the task goal, that is, participants could potentially complete our task without attending to the time intervals. Further, during learning, the cue was predictive of the associated CTI only if it was followed by the target stimulus. Nonmatching learning trials, in which the cue was followed by a nontarget probe stimulus, were shown with the nonassociated time interval. Thus, during learning the cue was predictive of the associated time interval only in 50% of the trials, thereby making the cue per se ineffective in predicting which CTI would be shown. We propose that time intervals became an implicit temporal associate to the cue–target pairs in associative memory. The cue-based retrieval of the temporal memory trace in turn guided attentional allocation to optimize recognition of the probe as the target item, similarly to how stimulus processing for nontarget items is optimized when it is associated in memory to the target item (Moores et al. 2003).

Recent studies showed hippocampal involvement in the temporal organization of discontinuous events during memory encoding and retrieval (Staresina and Davachi 2009; Schapiro et al. 2012; Hsieh et al. 2014). We predict that brain activity related to the implicit memory trace of time in the TPAT also includes the hippocampus. This prediction is supported by evidence that the hippocampus contains time cells that represent elapsed time, in which activity of multiple hippocampal cells are distributed across the time interval (Eichenbaum 2014). The ensemble of hippocampal cells and the sequence in which they become activated during retrieval differs for different temporally structured events in memory (MacDonald et al. 2011), suggesting that time cells provide a neural correlate for unique episodic events in memory. It currently remains an open question whether time cells play a role in hippocampal activity observed with fMRI. Our TPAT paradigm could be used to further investigate this issue by extending it to separate memory of elapsed time from memory for temporal order.

In conclusion, using a novel paradigm we show evidence for a memory of elapsed time, which can act as contextual retrieval cue to facilitate retrieval of sensory visual information uniquely associated to the time interval. The paradigm provides a means to further investigate how human memory encodes, stores, and retrieves time information.

## Supplementary Material

Supplemental Material
