# Supplementary material for: Learned interval time facilitates associate memory retrieval
Source: Learn Mem. 2017 Apr;24(4):158–61. doi: 10.1101/lm.044404.116 (PMC5362700; doi:10.1101/lm.044404.116)
Supplement: Supplemental Material [file supp_24_4_158__index.html]

Supplemental Material 

# Learned interval time facilitates associate memory retrieval

## Supplemental Material

- Supplemental\_Material.docx
