# Supplementary material for: Learned interval time facilitates associate memory retrieval
Source: Learn Mem. 2017 Apr;24(4):158–61. doi: 10.1101/lm.044404.116 (PMC5362700; doi:10.1101/lm.044404.116)
Supplement: Supplemental Material [file supp_24.4.158_Supplemental_Material.docx]

**SUPPLEMENTARY MATERIALS**

**Learnt interval time facilitates associate memory retrieval**

Vincent van de Ven, Sarah Kochs, Fren Smulders, Peter de Weerd

Department of Cognitive Neuroscience, Faculty of Psychology and Neuroscience, Maastricht University, The Netherlands

**Supplementary Methods**

*Participants*

For experiment 1, forty-one participants (36 females, 2 left-handed, mean [SD] age in years = 20.9 [1.8]) were recruited from the local student population of Maastricht University. For experiment 2, we recruited sixteen participants (all right-handed females, mean [SD] age = 20.5 [1.0]). All participants received financial compensation or course credit for participation, and provided written informed consent before participation. Study approval was obtained from the local ethical committee of the Faculty of Psychology and Neuroscience (FPN) of Maastricht University.

*Stimulus design and presentation*

We used two sets of sixteen coloured abstract shapes, which we generated using Fourier descriptors, using custom-written Matlab code that was adapted from freely available code (courtesy of John Myles White at <https://github.com/johnmyleswhite/FourierDescriptors>). The two sets differed in shape and color. **Figure S1** displays the sixteen shapes of one set. Each participant received one of the two stimulus sets for the task through random assignment.

Stimuli were shown centrally on a PC controlled computer screen with a refresh rate of 60 Hz at an eye-screen distance of approximately 57 cm, at a size of 5° x 5° visual angle. The onset of a trial was marked by the presentation of a white fixation cross for 500 msec, to which participants were instructed to fix their gaze on. Stimulus presentation timing and response collection were controlled using Psychopy version 1.8 (Peirce 2007), running on a Windows 7 operating system.

*Analysis*

The statistical analyses were performed in Matlab (version R2009b; Mathworks, Inc), using custom-written and publicly available routines (Trujillo-Ortiz et al. 2010; Vandekerckhove and Tuerlinckx 2008), and in the freely available software package JASP version 0.8.0.0 (JASP Team 2016).

*Use of the EZ-diffusion model*

A potential complication in the TPAT is that the systematic variations in time interval could alter responses independent of memory-related effects, such as decreasing response latencies with longer waiting periods (Näätänen 1971; Niemi and Näätänen 1981). Hence, it is possible that longer-lasting TPAT trials could lead to participants endorsing different response strategies for different temporal conditions. Analysing accuracy separately from response latencies provides an incomplete description and could thus obscure such confounding effects. To provide a more comprehensive description of recognition judgments in our task, we analysed the data not only according to response latencies and accuracy but also in terms of a diffusion model of decision-making, which combines distributions of response speed and accuracy to provide a latent variable description of decision-making parameters (Ratcliff 1978; Ratcliff and McKoon 2008; Voss et al. 2013; Wagenmakers et al. 2007). More specifically, diffusion models provide a latent variable description of the associations between the distribution of accuracy and reaction times, of which the *drift rate* (v), *boundary separation* (a) and *nondecision time* (Ter) are arguably the most relevant. Drift rate represents the quality of the information to make a decision, boundary separation refers to response conservativeness or the decision criterion at which a decision is made for one or the other alternative, and nondecision time refers to unspecific processes unrelated to the decision.

We used the EZ-diffusion model to estimate these three latent parameters (Wagenmakers et al. 2007), as it is algorithmically easy to use and requires only three input variables (proportion of response accuracy and response time mean and variance of correctly judged trials in seconds). Importantly, some authors have suggested that the algorithmic simplicity of the EZ-diffusion model may lead to biased or imprecise parameter estimates. Particularly, Ratcliff (Ratcliff 2008) demonstrated with a series of data simulations that the originally proposed EZ-diffusion model may give biased parameter estimates in the case of outliers in skewed reaction time distributions.

Wagenmakers and colleagues (Grasman et al. 2009; Wagenmakers et al. 2008) responded to these (and other) claims with an update to make the original EZ model more robust (termed “robust EZ” in (Wagenmakers et al. 2008)). The robust EZ updates are simple corrections to the RT mean and variance of the observed reaction time data (for details see (Wagenmakers et al. 2008)). Further, a recent analysis showed that the EZ model’s simplicity can provide comparable or even more statistical power in comparison to the full diffusion model under some circumstances (Ravenzwaaij et al. 2016).

Another consideration is that the ‘full’ diffusion model requires inputs about error distributions, which in turn requires a substantial amount of error trials. Also, the ‘full’ diffusion model is algorithmically complex, such that proper parameter estimation likewise depends on a substantial number of trials. The relatively few errors that participants made in our TPAT provide a challenge for parameters estimation with the ‘full’ diffusion model approaches but not for the EZ model (Wagenmakers et al. 2007; Ravenzwaaij et al. 2016).

In all, with these considerations we opted to use the EZ-diffusion model with the suggested “robust EZ” updates for corrected reaction time input parameters (i.e., RT mean and variance). Parameter estimates were obtained separately for each experimental condition and participant (see also (Ravenzwaaij et al. 2016)). We used the *ezdiff* function of the DMA Toolbox in Matlab for computation (Vandekerckhove and Tuerlinckx 2008), with additional custom-written code to implement the “robust EZ” corrections (see [www.ejwagenmakers.com](http://www.ejwagenmakers.com) or (van Ravenzwaaij and Oberauer 2009) for more details about available EZ diffusion code in various coding languages). We reported the robust EZ results in the main manuscript. We obtained highly similar results with the original EZ-diffusion model (not reported here).

**Supplementary Results**

*Experiment 1a and 1b*

To verify that the data of the two versions of Experiment 1 were similar, we first conducted a preliminary linear mixed effects model analysis. We included the between-subject factor Experiment (1a, 1b) and the within-subject factors Learnt CTI (500, 2000) and Tested CTI (500, 2000) and focused on the mixed effect results that included the between-subject factor. None of the mixed or between-subject effects were significant (see **Supplementary Table S1**). Therefore, we pooled the data from Experiments 1a and 1b (that is, collapsing the between-subject factor) to increase statistical power in a subsequent RMANOVA with within-subject factors Learnt CTI and Tested CTI.

We also verified that the interaction effect of sensitivity (d’, see main text) was similar for both Experiment 1a and 1b. Calculating separate RMANOVAs for Experiment 1a and 1b revealed a significant Learnt CTI x Tested CTI interaction effect for both (Experiment 1a: F[1,16] = 5.6, P = 0.031, ep^2 = 0.26; Experiment 1b: F[1,23] = 6.4, P = 0.019, ep^2 = 0.22).

*Diffusion model results*

We calculated separate RMANOVAs for drift rate, boundary separation and nondecision time, as estimated using the EZ-diffusion model (Wagenmakers et al. 2008, 2007). For drift rate *v*, we found a significant Learnt CTI x Tested CTI interaction effect (F[1,40] = 15.1, p < 0.001, ep^2 = 0.27), and no significant effects for Learnt CTI (F[1,40] = 0.5, P = 0.49) and Tested CTI (F[1,40] = 0.8, P = 0.38). The pattern of the interaction was highly similar to the interaction pattern of accuracy (see **Figure 2** of the main text). Posthoc comparisons showed higher *v* for cue-target pairs that were learnt with a short CTI when these pairs were also tested with the short CTI (mean [SE] *v* = 0.12 [0.01]), compared to when tested with the long CTI (0.11 [0.01]; t[40] = 2.8, p = 0.007, Cohen’s d = 0.44). A similar effect was found when pairs learnt with the long CTI were tested with the long (0.13 [0.01]) vs. short interval (0.11 [0.01]; t[40] = -3.5, p = 0.001, Cohen’s d = 0.54).

No significant main or interaction effects were found for boundary separation (Learnt CTI: F[1,40] = 1.5, P = 0.23; Tested CTI: F[1,40] = 0.7, P = 0.42; Learnt CTI x Tested CTI: F[1,40] = 3.9, P = 0.06) or nondecision time (Learnt CTI: F[1,40] = 0.9, P = 0.36; Tested CTI: F[1,40] = 0.2, P = 0.64; Learnt CTI x Tested CTI: F[1,40] = 0.2, P = 0.66), indicating that participants showed no CTI-dependent changes in response bias or response delays. In summary, these findings indicate that recognition decisions were made more easily for those trials in which the presented CTI matched the learnt cue-dependent CTI, corroborating the results of the previous analyses of response time and accuracy (see main text).

*Experiment 2 null-results*

We further quantified the null-results of Experiment 2 using a Bayesian version of the RMANOVA hypothesis test reported in the main text. Contrary to null-hypothesis significance testing, Bayesian analysis allows quantification of evidence in favor of *H*1 or *H*0, in the form of a Bayes Factor, *B*_10_ (see for example (Wagenmakers 2007; Jarosz and Wiley 2014)). A *B*_10_ > 1 indicates evidence in favor of *H*1, *B*_10_ = 1 indicates no evidence and *B*_10_ < 1 indicates evidence in favor of *H*0. Bayesian analyses were performed using JASP (JASP Team 2016). **Supplementary Table S4** lists Bayes Factors *B*_10_ for all main and interaction effects that were analysed using RMANOVA (see main text and **Supplementary Table S3**). Results show that the data of Experiment 2 are more likely to occur under *H*0 than under *H*1, thereby corroborating the results reported in the main text.

**Supplementary Tables**

**Table S1. Linear mixed-effects for Experiment 1.** Mixed-effects ANOVA included between-subject factor Experiment (1a, 1b) and within-subject factors Learnt CTI (500, 2000) and Tested CTI (500, 2000). The table only reports effects that include the between-subject factor Experiment, with degrees of freedom [1, 39].

*Factor* F P

Experiment 1.9 0.18

Experiment x Learnt CTI 0.1 0.82

Experiment x Tested CTI 0.2 0.64

Experiment x Learnt CTI x Tested CTI 0.0 0.99

**Table S2. RMANOVA values for d’ and reaction times of Experiment 1.** Degrees of freedom for F-tests are [1, 40] for each of the factors.

*Factor* d’ Reaction times

F P F P

Learnt CTI 0.6 0.46 2.6 0.12

Tested CTI 0.2 0.69 6.4 0.02

Learnt CTI x Tested CTI 12.2 0.001 1.5 0.23

**Table S3. RMANOVA values for d’ and reaction time of Experiment 2.** Degrees of freedom for F-tests are [1, 15] for each of the factors.

*Factor* d’ Reaction times

F P F P

Learnt CTI 1.0 0.34 0.0 0.84

Tested CTI 0.0 0.89 2.2 0.16

Learnt CTI x Tested CTI 1.8 0.20 0.0 0.88

**Table S4. Characterizing Experiment 2 null-results using Bayes Factors, *B*_10_.** Compare 1-Factor model results in main text and 2-Factor model results with Supplementary Table S3.

*Model* Factor *B*_10_ Qualification

*1-Factor* Test CTI 0.36 Evidence in favor of *H*0

*2-Factor* Learn CTI 1.04 No evidence

Test CTI 0.25 Evidence in favor of *H*0

Learn CTI x Test CTI 0.11 Evidence in favor of *H*0

**Supplementary Figures**

**Figure S1. Abstract shapes.** Shown are the sixteen stimuli of one of the stimulus sets used in the experiments. The sixteen stimuli are organized in eight pairs. Four pairs are associated to a cue-target interval (CTI) of 500 msec and the other four pairs to a CTI of 2000 msec.

**Supplementary References**

Grasman RPPP, Wagenmakers E-J, Van Der Maas HLJ. 2009. On the mean and variance of response times under the diffusion model with an application to parameter estimation. *J Math Psychol* **53**: 55–68.

Jarosz AF, Wiley J. 2014. What are the odds? A practical guide to computing and reporting Bayes Factors. *J Probl Solving* **7**: 2–9.

JASP Team. 2016. JASP.

Näätänen R. 1971. Non-aging fore-periods and simple reaction time. *Acta Psychol (Amst)* **35**: 316–327.

Niemi P, Näätänen R. 1981. Foreperiod and simple reaction time. *Psychol Bull* **89**: 133–162.

Peirce JW. 2007. PsychoPy-Psychophysics software in Python. *J Neurosci Methods* **162**: 8–13.

Ratcliff R. 1978. A theory of memory retrieval. *Psychol Rev* **85**: 59–108.

Ratcliff R. 2008. The EZ diffusion method: too EZ? *Psychon Bull Rev* **15**: 1218–28.

Ratcliff R, McKoon G. 2008. The diffusion decision model: theory and data for two-choice decision tasks. *Neural Comput* **20**: 873–922.

Ravenzwaaij D Van, Donkin C, Vandekerckhove J. 2016. The EZ Diffusion Model Provides a Powerful Test of Simple Empirical Effects. *Psychon Bull Rev* 1–14.

Trujillo-Ortiz A, Hernandez-Walls R, Trujillo-Perez FA. 2010. RMAOV33: Three-way Analysis of Variance With Repeated Measures on Three Factors Test. A MATLAB file. *Matlab Cent Fileexchange*. http://www.mathworks.com/matlabcentral/fileexchange/loadFile.do?objectId=9638.

van Ravenzwaaij D, Oberauer K. 2009. How to use the diffusion model: Parameter recovery of three methods: EZ, fast-dm, and DMAT. *J Math Psychol* **53**: 463–473.

Vandekerckhove J, Tuerlinckx F. 2008. Diffusion model analysis with MATLAB: a DMAT primer. *Behav Res Methods* **40**: 61–72.

Voss A, Nagler M, Lerche V. 2013. Diffusion models in experimental psychology: A practical introduction. *Exp Psychol* **60**: 385–402.

Wagenmakers E-J. 2007. A practical solution to the pervasive problems of p values. *Psychon Bull Rev* **14**: 779–804.

Wagenmakers E-J, van der Maas HLJ, Dolan C V, Grasman RPPP. 2008. EZ does it! Extensions of the EZ-diffusion model. *Psychon Bull Rev* **15**: 1229–1235.

Wagenmakers E-J, van der Maas HLJ, Grasman RPPP. 2007. An EZ-diffusion model for response time and accuracy. *Psychon Bull Rev* **14**: 3–22.
